# Verification of reference intervals for routine biochemical tests using the reflimR in Turkish adults

**DOI:** 10.1371/journal.pone.0342530

**Published:** 2026-02-11

**Authors:** Levent Deniz, Ozlem Demirelce

**Affiliations:** 1 Department of Medical Biochemistry, University of Health Sciences Türkiye, Istanbul Training and Research Hospital, Istanbul, Türkiye; 2 Department of Medical Biochemistry, Istanbul Goztepe Prof. Dr. Suleyman Yalcin City Hospital, Istanbul, Türkiye; Tribhuvan University Institute of Medicine, NEPAL

## Abstract

This study aimed to verify the reference interval(s) (RIs) for routine biochemistry tests in adult Turkish population using the reflimR method and compare them with the manufacturer-provided RIs. The RIs of 19 routine biochemical parameters, analyzed using Beckman Coulter analyzers between February and October 2024, were evaluated using the reflimR algorithm. The RIs were estimated separately for females and males using five indirect approaches (reflimR, refineR, KOSMIC, Hoffmann, and Bhattacharya). A traffic light algorithm based on permissible uncertainty was used to interpret whether the RIs limits calculated with reflimR were within the tolerance limits. Using reflimR, 40 of 76 RI limits were accepted, 21 required checking, and 15 were rejected for RI verification compared with the manufacturer-provided values. The comparison of reflimR with other indirect methods generally produced concordant results, except for the alanine aminotransferase (ALT), gamma-glutamyl transferase (GGT), and total bilirubin tests. The reflimR algorithm may offer a swift and accessible method for calculating and verifying RIs. Verification failures may arise from fundamental variations, including ethnicity, sex, age demographics, and geographic factors, between the manufacturer’s study results and our analyzed population.

## Introduction

Reference interval(s) (RIs) are one of the most common decision-support tools used to interpret numerical values in clinical laboratory reports. Laboratory results are interpreted by comparing them with these intervals; therefore, the quality of RIs can be as important as that of the results when making accurate clinical decisions [1]. The classical approach to determining RIs involves collecting samples from a reference population of at least 120 healthy individuals and calculating RIs as specified percentiles (e.g., 2.5^th^ and 97.5^th^ percentiles) using the direct method. Many RIs require appropriate stratification because they depend on variables such as ethnicity, sex, age, geographic region of residence, diet, and exercise. Therefore, laboratories must generate their own RIs or validate existing ones [2]. An alternative approach is the indirect approach, in which the results of routine samples are used to determine RIs. The indirect method has some important advantages over direct methods, such as being faster and less expensive. The disadvantages of indirect methods include the possible influence of unhealthy (diseased) subpopulations on the derived range [3]. In conclusion, any method used to determine the RIs between the distributions of healthy and diseased individuals must be applied with accuracy and high precision. Several indirect techniques have been developed and are routinely used for RI estimation. Important approaches that have been applied in the past include visual Hoffman [4] and Bhattacharya methods [5]. Recently, more sophisticated indirect methods based on open-source software algorithms have been developed, such as the truncated maximum likelihood (TML) method developed by Arzideh et al. [6] and the truncated minimum chi-square method (TMC) developed by Wosniok et al. [7], the kolmogorov-smirnov distance (KOSMIC) (an updated version of the TML technique) by Zierk et al. [8], and most recently, refineR by Ammer et al. [9].

An alternative R package called reflimR, developed by Hoffman et al., has been proposed as a tool that can provide results in a shorter time and perform more accurate and precise calculations for results below 1000. To achieve the intended purpose with this tool, an algorithm was added to the package, and it was possible to observe how well the estimated RIs matched the predefined limits used in the laboratory using traffic light colors. The reflimR algorithm facilitates RIs verification by employing the manufacturer’s or literature values’ specified tolerance limits [10].

This study aimed to verify the RIs for routine biochemistry tests in adult patients using the reflimR method and to compare them with the RIs transferred from the values provided by the manufacturer. Additionally, we investigated whether the RIs obtained using the reflimR method were consistent with those obtained using other indirect algorithms, including refineR, KOSMIC, Hoffmann, and Bhattacharya.

## Materials and methods

Routine biochemistry test results were collected from the Biochemistry Department of Istanbul Training and Research Hospital via the laboratory information system (Alis, Ventura Software, Ankara, Türkiye) between 01/02/2024 and 31/10/2024. Data were accessed for research purposes on 01/02/2025. This study was conducted with the approval of the Clinical Research Ethics Committee of Istanbul Training and Research Hospital (date: 25.01.2025, number:17) and in accordance with the principles of the Declaration of Helsinki. Since this study was designed as a retrospective analysis based on medical records, all data were fully anonymized prior to access. The ethics committee waived the requirement for informed consent owing to the retrospective nature of the study.

### Instruments and reagents

Serum thyroid-stimulating hormone (TSH), free triiodothyronine (FT3), and free thyroxine (FT4) levels were measured using a chemiluminescence method with a DxI 800 analyzer (Beckman Coulter Inc., Brea, CA, US). Serum glucose, total protein, albumin, alanine aminotransferase (ALT), aspartate aminotransferase (AST), gamma-glutamyl transferase (GGT), lactate dehydrogenase (LDH), total bilirubin, calcium, magnesium, phosphorus, urea, creatinine, sodium, potassium, and chloride levels were measured using an AU5800 analyzer (Beckman Coulter Inc., Brea, CA, US). The detailed assay methods for the analytes are provided in Table 1. The instruments remained stable throughout the study period. The analyzer was maintained according to the manufacturer’s instructions, and its analytical stability was evaluated over time. Calibration and maintenance were performed using the original products provided by Beckman Coulter^®^. Internal quality control and external quality assessment tests consistently met the desired analytical performance targets. The manufacturer-provided RIs supplied by Beckman Coulter^®^ were originally established on populations from North America and Western Europe. These RIs were inserted with local verification throughout the implementation of the analyzer.

**Table 1:** Assay methods for the relevant analytes on AU 5800 for biochemical tests and DxI 800 for hormone tests.

Analyte	Unit	Method	Method description	AMR
Glucose	mg/dL	Hexokinase	Enzymatic UV test (hexokinase)	10-810
Albumin	g/L	Bromocresol Green dye binding	Photometric colour test	15-60
TP	g/L	Biuret	Photometric colour test	30-120
ALT	U/L	NADH (without P-5′-P)	IFCC reccommended method	3-500
AST	U/L	NADH (without P-5′-P)	IFCC reccommended method	3−1,000
GGT	U/L	Gamma-glutamyl-3-carboxy-4-nitroanilide	IFCC reccommended method	5−1,200
LDH	U/L	Lactate to Pyruvate	IFCC reccommended method	25−1,200
TB	mg/dL	Diazonium salt	Photometric	0.03-30
Ca	mg/dL	Arsenazo III dye	Photometric colour test	4-20
Mg	mg/dL	Xylidyl blue	Photometric colour test	0.5-8
P	mg/dL	Phosphomolybdate complex	Photometric UV test	1-20
Urea	mg/dL	Urease/GLDH	Kinetic UV test	5-300
Creatinine	mg/dL	Modified kinetic Jaffè	Kinetic Jaffe (compensated method)	0.06-25
Sodium	mmol/L	Ion selective electrode/ diluted (indirect)	Ion selective indirect method	50-200
Potassium	mmol/L	Ion selective electrode/ diluted (indirect)	Ion selective indirect method	1-10
Chloride	mmol/L	Ion selective electrode/ diluted (indirect)	Ion selective indirect method	50-200
TSH	mIU/L	Two-site sandwich immunoassay (3^rd^ IS)	Chemiluminescent immunoassay	0.005-50.0
FT4	ng/dL	Two-step competitive immunoassay	Chemiluminescent immunoassay	0.25–6.0
FT3	pg/mL	Two-step competitive immunoassay	Chemiluminescent immunoassay	0.88–30

AMR, analytical measurement range; TP, Total Protein; ALT, Alanine Aminotransferase; AST, Aspartate Aminotransferase; GGT, Gamma-Glutamyl Transferase; LDH, Lactate Dehydrogenase; TB, Total Bilirubin; Ca, Calcium; Mg, Magnesium; P, Phosphorus; TSH, Thyroid Stimulating Hormone; FT4, Free Thyroxine; FT3, Free Triiodothyronine.

### Data cleaning

Data pre-processing was performed using Excel (Microsoft, Redmond, WA, US). The data obtained were filtered prior to analysis. During the study period, each analyte was extracted separately from the laboratory information system as an independent dataset. Therefore, the male and female sample sizes were analyte-specific and may not represent matched patient cohorts across different tests. Non-numeric or rejected sample results were excluded. For individuals with multiple laboratory test results, only the initial results were considered based on their identification numbers, assuming that the necessity of multiple tests indicated a higher likelihood of a pathological condition, and this approach was applied consistently across all analytes. Results from intensive care units, hematology, oncology, nephrology (hemodialysis), infectious diseases, gastroenterology, endocrinology, obesity, interventional radiology, nuclear medicine, and radiation oncology departments were excluded. Individuals aged <18 and >75 years were excluded from the study.

### Statistical analysis

Statistical analyses were performed using R Studio (version 4.4.1) and SPSS (version 26; IBM Corp.). Bland-Altman analyses were performed using MedCalc® Statistical Software version 23.4.5 (MedCalc Software Ltd, Ostend, Belgium; https://www.medcalc.org; 2025). The normality of the data was evaluated using the Kolmogorov-Smirnov test. RIs were estimated separately for females and males using five indirect approaches (reflimR, refineR, KOSMIC, Hoffmann, and Bhattacharya). RefineR method was employed to estimate RIs using the web-based interface at https://kc.uol.de/rifindr/. The KOSMIC method was employed to estimate RIs using the web-based interface at https://KOSMIC.diz.uk-erlangen.de. Hoffmann analysis was conducted using this website (https://gocrunch.shinyapps.io/HoffApp/). Bhattacharya analysis was conducted using the website (https://gocrunch.shinyapps.io/BhattApp/). The lower and upper reference limits (LRLs and URLs) of the RIs were calculated as the 2.5th and 97.5th percentiles, respectively. The reference limits obtained using the reflimR algorithm were compared with those derived from other indirect methods by calculating absolute or percentage differences. These differences were then evaluated against the allowable total error limits defined by the Clinical Laboratory Improvement Amendments (CLIA) (https://westgard.com/clia-a-quality/quality-requirements/2024-clia-requirements.html). Because the CLIA performance goals for calcium, sodium, and potassium are specified in absolute units, the differences for these analytes were calculated and interpreted as absolute values. For the remaining analytes, the differences were expressed as percentages.

### reflimR

**Fig 1:**
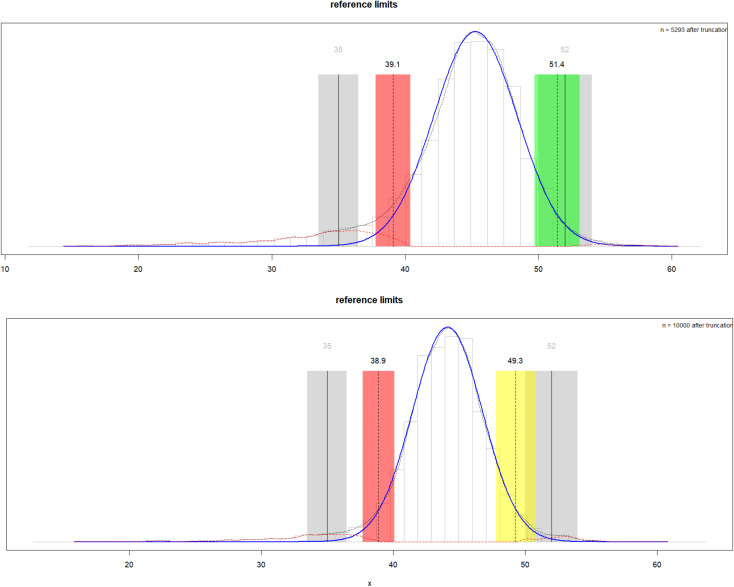
Traffic light algorithm-based comparison of albumin reference limits with reflimR. The upper panel illustrates the albumin results for male participants and the lower panel presents the corresponding albumin results for female participants. The figure displays the estimated reference limits (dashed lines), medical tolerance limits (gray bars), theoretical reference distribution (blue curve), and pathological outlier distributions (red curves). Accordingly, reflimR outputs are classified as ‘within tolerance’ (green), ‘slightly increased/decreased’ (yellow), and ‘markedly increased/decreased’ (red).

The reflimR method [10], known as the “modified Hoffmann” approach, is valued because of its simplicity and low computational requirements. It builds on the original probability paper method by incorporating Bowley’s quartile skewness to determine whether a normal or lognormal distribution is appropriate, applying log transformation if necessary, truncating the central 95% of non-pathological values using the iBoxplot95 algorithm, and calculating the reference limits from a normal quantile-quantile plot. It can be downloaded from GitHub and installed in the R environment following the instructions provided on the website: https://github.com/SandraKla/reflimR_Shiny (accessed on 10/02/2025). The reflimR package includes several functions, with reflim(x) serving as the main function. It calls the other important functions that can be arranged in three groups: Group 1 includes the main functions that provide the user with the final results of reflim() and help to interpret them: ri_hist() creates a graphical output (histogram with RI and density curves), permissible_uncertainty() calculates the medical tolerance limits of the results, and interpretation() assesses the medical significance of deviations from given target values. Group 2 performs the three underlying statistical operations of modelling, truncation, and calculation, and group 3 contains auxiliary functions for miscellaneous tasks. The reflim function can be executed by simply calling reflim(x), where x is a vector of numeric data to be analyzed. As shown in Fig 1, the main result of the reflim function is an illustrative graphic showing a histogram of the original data with a dotted overall density curve. The estimated reference limits are displayed as dashed vertical lines and the respective medical tolerance limits as gray bars. The blue solid density curve represents the theoretical distribution of the assumed reference population, and the density curves of potential pathological outliers are shown in red. For each analyte, the RI values derived from the reflimR algorithm were compared with those obtained from the permissible uncertainty (tolerance limits) algorithm, values reported in the instructions for use (IFU) or the literature, and RIs generated using other data mining algorithms. The green bars indicate that the predicted target values were within the tolerance ranges determined by reflimR. Yellow bars signify that the target values are outside the tolerance range but still have some overlap, whereas red bars indicate that there is no overlap in the tolerance ranges. Accordingly, the outputs from the reflimR function are categorized as “within tolerance” (green), “slightly increased/decreased” (yellow), and “markedly increased/decreased” (red). In the reflimR package, 95% confidence intervals were calculated using the conf_int95 function, which estimates the interval through 100,000 Monte Carlo simulations [10]. As the manufacturer-provided RIs for ALT, AST, GGT, and LDH did not include LRLs, a comparison with the reflimR results was not feasible; therefore, reference limits from the literature were used [11].

## Results

The detailed age distribution, sample size per analyte, and sex are provided in Table 2. The total number of patient measurements analyzed for all 19 biochemical parameters was 694,869. Across the 19 analytes and two sex-specific subgroups (38 subgroups in total), the median sample size was 15,600, with a range of 3,772–63,266 measurements. The total sample sizes across the 19 analytes showed a median of 26,355 measurements, with a range of 10,195–93,561. Among the sex-specific subgroups, the female sample sizes ranged from 6,181–63,266 (median = 16,715), whereas the male sample sizes ranged from 3,772–30,295 (median = 9,640). The study cohort reflects the broader adult demographic residing in Istanbul, an ethnically heterogeneous metropolitan area primarily composed of individuals of Turkish origin.

**Table 2:** Verification of RIs for adults aged 18-75 with the reflimR algorithm for laboratory parameters.

Analyte	Unit	N	Age	Sex	IFU RI	RI	95%CI LL	95%CI UL	Tolerance Status^b^	
Glucose	mg/dL	20,683	52 (38-62)	M	74.0-106	77.2-119	76.9-77.6	118-119	**LL**	**UL**
Glucose	mg/dL	32,513	49 (35-60)	F	74.0-106	74.6-112	74.3-75.0	112−112	**LL**	**UL**
Albumin	g/L	6,063	51 (38-62)	M	35.0-52.0	39.1-51.4	38.9-39.4	51.1-51.6	**LL**	**UL**
Albumin	g/L	10,969	51 (39-61)	F	35.0-52.0	38.9-49.3	38.8-39.1	49.1-49.4	**LL**	**UL**
TP	g/L	3,949	52 (38-62)	M	66.0-83.0	63.5-83.6	63.0-64.2	82.9-84.1	**LL**	**UL**
TP	g/L	6,372	50 (37-61)	F	66.0-83.0	62.9-83.4	62.5-63.4	82.9-83.8	**LL**	**UL**
ALT	U/L	25,099	52 (38-62)	M	9.00-57.0^a^	8.30-50.3	8.16-8.48	49.2-51.2	**LL**	**UL**
ALT	U/L	39,033	49 (35-60)	F	7.00-28.0^a^	7.30-30.4	7.22-7.40	29.9-30.7	**LL**	**UL**
AST	U/L	20,240	51 (37-61)	M	13.0-30.0^a^	13.6-36.2	13.5-13.8	35.7-36.6	**LL**	**UL**
AST	U/L	34,301	48 (33-59)	F	11.0-25.0^a^	12.7-31.4	12.6-12.8	31.1-31.6	**LL**	**UL**
GGT	U/L	6,484	50 (35-61)	M	11.0-69.0^a^	10.6-64.7	10.3-11.1	61.9-66.9	**LL**	**UL**
GGT	U/L	11,352	49 (33-60)	F	7.00-33.0^a^	8.00-33.5	7.84-8.21	32.6-34.2	**LL**	**UL**
LDH	U/L	6,972	54 (42-64)	M	126-220^a^	119-240	118-121	236-243	**LL**	**UL**
LDH	U/L	9,179	52 (40-63)	F	126-220^a^	117-250	116-119	246-253	**LL**	**UL**
TB	mg/dL	4,014	51 (36-61)	M	0.30-1.20	0.31-1.46	0.30-0.33	1.39-1.52	**LL**	**UL**
TB	mg/dL	6,181	49 (36-60)	F	0.30-1.20	0.26-1.02	0.25-0.27	0.98-1.04	**LL**	**UL**
Ca	mg/dL	7,811	52 (38-62)	M	8.80-10.6	9.08-10.7	9.05-9.12	10.7-10.7	**LL**	**UL**
Ca	mg/dL	14,485	51 (39-61)	F	8.80-10.6	9.18-10.5	9.16-9.20	10.4-10.5	**LL**	**UL**
Mg	mg/dL	5,273	50 (36-61)	M	1.70-2.30	1.72-2.27	1.71-1.74	2.25-2.28	**LL**	**UL**
Mg	mg/dL	12,237	50 (38-61)	F	1.70-2.30	1.70-2.25	1.69-1.71	2.24-2.26	**LL**	**UL**
P	mg/dL	3,772	51 (38-61)	M	2.50-4.60	2.51-4.68	2.47-2.56	4.58-4.75	**LL**	**UL**
P	mg/dL	7,943	52 (40-62)	F	2.50-4.60	2.73-4.60	2.70-2.77	4.56-4.63	**LL**	**UL**
Urea	mg/dL	23,671	52 (39-62)	M	17.0-43.0	18.5-52.8	18.3-18.7	52.1-53.3	**LL**	**UL**
Urea	mg/dL	35,864	49 (36-60)	F	17.0-43.0	14.7-48.9	14.6-14.9	48.3-49.4	**LL**	**UL**
Creatinine	mg/dL	20,622	51 (37-62)	M	0.67-1.17	0.67-1.22	0.66-0.67	1.21-1.23	**LL**	**UL**
Creatinine	mg/dL	34,567	49 (36-60)	F	0.51-0.95	0.50-0.94	0.50-0.51	0.93-0.94	**LL**	**UL**
Sodium	mmol/L	18,677	53 (40-63)	M	136-146	136-144	136−136	144−144	**LL**	**UL**
Sodium	mmol/L	28,471	49 (36-60)	F	136-146	136-144	136−136	144−144	**LL**	**UL**
Potassium	mmol/L	18,031	53 (39-63)	M	3.50-5.10	3.80-4.98	3.79-3.82	4.96-4.99	**LL**	**UL**
Potassium	mmol/L	27,816	49 (36-60)	F	3.50-5.10	3.82-4.99	3.81-3.83	4.98-5.00	**LL**	**UL**
Chloride	mmol/L	7,150	56 (44-65)	M	101-109	98.0-108	97.8-98.2	108−108	**LL**	**UL**
Chloride	mmol/L	8,124	55 (44-64)	F	101-109	99.0-108	98.9-99.2	108−108	**LL**	**UL**
TSH	mIU/L	30,295	50 (36-61)	M	0.38-5.33	0.60-4.95	0.59-0.61	4.84-5.04	**LL**	**UL**
TSH	mIU/L	63,266	46 (33-58)	F	0.38-5.33	0.64-6.21	0.63-0.65	6.11-6.29	**LL**	**UL**
FT4	ng/dL	18,129	52 (38-62)	M	0.61-1.12	0.62-1.14	0.62-0.63	1.13-1.15	**LL**	**UL**
FT4	ng/dL	38,906	48 (35-59)	F	0.61-1.12	0.60-1.15	0.59-0.60	1.14-1.16	**LL**	**UL**
FT3	pg/mL	9,640	54 (43-64)	M	2.50-3.90	2.46-4.16	2.43-2.49	4.13-4.19	**LL**	**UL**
FT3	pg/mL	16,715	51 (38-61)	F	2.50-3.90	2.39-3.97	2.37-2.41	3.95-3.99	**LL**	**UL**

N, Numbers; IFU, Instructions for Use (Beckman Coulter^®^); RI, Reference Interval (2.5^th^ and 97.5^th^ percentiles); LL, Lower Limit; UL, Upper Limit; TP, Total Protein; ALT, Alanine Aminotransferase; AST, Aspartate Aminotransferase; GGT, Gamma-Glutamyl Transferase; LDH, Lactate Dehydrogenase; TB, Total Bilirubin; Ca, Calcium; Mg, Magnesium; P, Phosphorus; TSH, Thyroid Stimulating Hormone; FT4, Free Thyroxine; FT3, Free Triiodothyronine. ^a^ [11]; ^b^ green: accept = within tolerance; yellow: check = slightly increase/decreased; red: reject = markedly increase/decreased.

Table 2 presents the verification results of manufacturer-recommended RIs for 19 routine biochemical tests using the reflimR algorithm in women and men aged 18–75 years. In the male population, the URLs for the glucose, AST, total bilirubin, and urea tests and the LRLs for the albumin, potassium, and TSH tests markedly exceeded the established tolerance limits based on the reflimR algorithm. In women, the URLs for AST, LDH, total bilirubin, and urea tests and the LRLs for albumin, AST, potassium, and TSH also markedly exceeded the tolerance limits based on the reflimR algorithm. Fifteen of the 76 RI limits were rejected (“red: markedly increase/decreased”) for RI verification. In the male population, the URLs for ALT, LDH, and FT3, as well as the LRLs for glucose, total protein, LDH, calcium, urea, and chloride tests, were found to slightly exceed the established tolerance limits based on the reflimR algorithm. In women, the URLs for glucose, albumin, ALT, and TSH and the LRLs for total protein, GGT, LDH, total bilirubin, calcium, phosphorus, urea, and chloride slightly exceeded the tolerance limits based on the reflimR algorithm. Twenty-one of the 76 RI limits required further evaluation (“yellow = slightly increased/decreased”) for RI verification. Forty of the 76 RI limits were accepted (“green= within tolerance”) for RI verification. For both sexes, the URLs and LRLs for magnesium, creatinine, sodium, and FT4 were within tolerance limits (Table 2).

**Fig 2:**
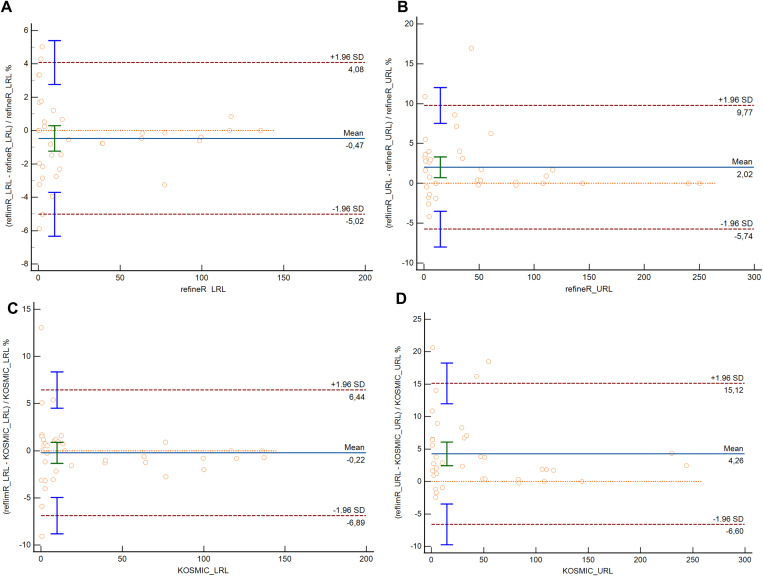
Bland–Altman plots comparing the lower and upper reference limits estimated by reflimR with those estimated by refineR and KOSMIC. The solid horizontal line represents the mean difference, whereas the dashed lines indicate the 95% limits of agreement (mean ± 1.96 SD).

**Fig 3:**
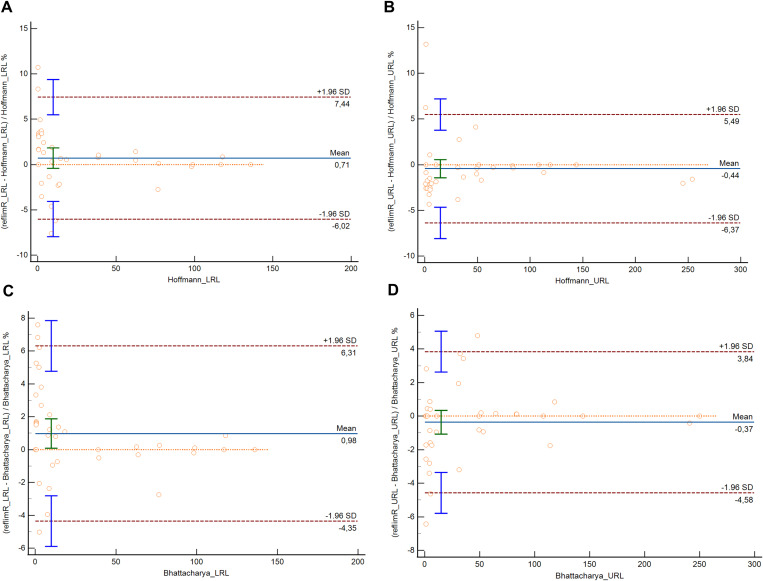
Bland–Altman plots comparing lower and upper reference limits estimated by reflimR with those estimated by Hoffmann and Bhattacharya. The solid horizontal line represents the mean difference, whereas the dashed lines indicate the 95% limits of agreement (mean ± 1.96 SD).

Table 3 presents the RIs calculated using the refineR, KOSMIC, Hoffmann, and Bhattacharya algorithms, as well as reflimR for sex-specific subgroups of 19 parameters. The percentage or absolute differences between the reference limits estimated by reflimR and those derived from other indirect methods are presented in Table 4. The URLs calculated using the reflimR algorithm were slightly higher for ALT (8.57%), AST (7.17%), total bilirubin (10.9%), and FT4 (5.50%) in females than those calculated using the refineR algorithm. Compared to the refineR algorithm, the LRL calculated by the reflimR algorithm was slightly lower for FT3 (−5.02%) in males and slightly higher for magnesium (4.29%) in females. The URL calculated using the reflimR algorithm for ALT (16.9%) in males was markedly higher than that calculated using the refineR algorithm. The URLs calculated using the reflimR algorithm were slightly higher for AST (7.10% in males, 8.28% in females), TSH (14.1% in males, 8.95% in females), FT4 (6.54% in males, 6.48% in females) in both sexes, ALT (16.2%), calcium (0.30 mg/dL), chloride (1.89%) in males, total bilirubin (10.9%), creatinine (5.62%) in females than those calculated using the KOSMIC algorithm. Compared with the KOSMIC algorithm, the LRL calculated by the reflimR algorithm was slightly lower for chloride (−2.00%) in males, whereas it was slightly higher for total bilirubin (13.0%) in females and FT4 (5.08%) in males. The URLs calculated using the reflimR algorithm were markedly higher for GGT (18.5%) and total bilirubin (20.7%) levels in males than those calculated using the KOSMIC algorithm. Compared with the Hoffmann algorithm, the LRL calculated by the reflimR algorithm was slightly higher for total bilirubin (10.7%) in males and magnesium (4.94%) in females. The URL calculated using the reflimR algorithm was slightly higher for total bilirubin (13.2%) in males than that calculated using the Hoffmann algorithm. Compared with the Bhattacharya method, the LRL for magnesium (6.83%) in males, phosphorus (6.23%) and magnesium (7.59%) in females calculated using the reflimR algorithm were found to be slightly higher. Compared with the Bhattacharya method, the LRL for FT3 (−5.02%) in males using the reflimR algorithm was found to be slightly lower (Tables 3 and 4). In the Bland–Altman analysis, the mean differences were −0.47% and 2.02% for the LRLs and URLs, respectively, in the comparison between reflimR and refineR, and −0.22% and 4.26% for the corresponding comparison between reflimR and KOSMIC (Fig 2). Similarly, the mean differences were 0.71% and −0.44% for the LRLs and URLs in the comparison between reflimR and the Hoffmann method, and 0.98% and −0.37% for the corresponding comparison between reflimR and the Bhattacharya method (Fig 3).

**Table 3:** Reference intervals derived from refineR, KOSMIC, Hoffmann, and Bhattacharya for laboratory parameters.

Analyte	Unit	Sex	reflimR		refineR		KOSMIC		Hoffmann		Bhattacharya	
			LL	UL	LL	UL	LL	UL	LL	UL	LL	UL
Glucose	mg/dL	M	77.2	119	77.3	117	76.5	117	77.1	119	77.0	118
Glucose	mg/dL	F	74.6	112	77.1	111	76.7	110	76.7	113	76.7	114
Albumin	g/L	M	39.1	51.4	39.4	51.2	39.5	51.2	38.7	51.4	39.3	51.3
Albumin	g/L	F	38.9	49.3	39.2	49.4	39.4	49.1	38.6	49.5	38.9	49.3
TP	g/L	M	63.5	83.6	63.6	83.5	64.3	83.8	62.6	83.9	63.7	83.5
TP	g/L	F	62.9	83.4	63.2	83.6	63.3	83.1	62.6	83.5	62.8	83.3
ALT	U/L	M	8.30	50.3	8.64	43.0	8.21	43.3	8.70	48.3	8.50	48.0
ALT	U/L	F	7.30	30.4	7.36	28.0	7.53	29.7	7.40	31.6	7.60	31.4
AST	U/L	M	13.6	36.2	13.8	35.1	13.5	33.8	13.9	36.7	13.7	35.0
AST	U/L	F	12.7	31.4	13.0	29.3	12.5	29.0	13.0	31.5	12.6	30.8
GGT	U/L	M	10.6	64.7	10.9	60.9	10.5	54.6	11.3	64.9	10.7	64.6
GGT	U/L	F	8.00	33.5	8.12	32.2	7.59	31.4	8.66	32.6	7.93	32.3
LDH	U/L	M	119	240	118	240	120	230	118	245	118	241
LDH	U/L	F	117	250	117	250	117	244	117	254	117	250
TB	mg/dL	M	0.31	1.46	0.30	1.42	0.32	1.21	0.28	1.29	0.30	1.42
TB	mg/dL	F	0.26	1.02	0.26	0.92	0.23	0.92	0.24	0.96	0.26	1.09
Ca	mg/dL	M	9.08	10.7	9.07	10.7	9.28	10.4	9.05	10.7	8.97	10.7
Ca	mg/dL	F	9.18	10.5	9.07	10.7	9.07	10.6	9.01	10.7	8.99	10.6
Mg	mg/dL	M	1.72	2.27	1.69	2.28	1.71	2.21	1.66	2.33	1.61	2.27
Mg	mg/dL	F	1.70	2.25	1.63	2.26	1.68	2.23	1.62	2.29	1.58	2.24
P	mg/dL	M	2.51	4.68	2.39	4.50	2.54	4.51	2.42	4.63	2.39	4.64
P	mg/dL	F	2.73	4.60	2.79	4.80	2.71	4.68	2.64	4.67	2.57	4.64
Urea	mg/dL	M	18.5	52.8	18.6	51.9	18.8	50.9	18.4	53.7	18.3	53.3
Urea	mg/dL	F	14.7	48.9	14.6	48.7	14.7	47.1	14.6	49.4	14.5	49.3
Creatinine	mg/dL	M	0.67	1.22	0.67	1.20	0.66	1.20	0.65	1.22	0.66	1.22
Creatinine	mg/dL	F	0.50	0.94	0.51	0.91	0.50	0.89	0.50	0.96	0.50	0.94
Sodium	mmol/L	M	136	144	136	144	137	144	136	144	136	144
Sodium	mmol/L	F	136	144	136	144	136	144	136	144	136	144
Potassium	mmol/L	M	3.80	4.98	3.79	5.05	3.78	4.92	3.71	5.11	3.70	4.96
Potassium	mmol/L	F	3.82	4.99	3.80	4.95	3.83	4.89	3.77	5.10	3.68	5.07
Chloride	mmol/L	M	98.0	108	98.6	108	100	106	98.2	108	98.2	108
Chloride	mmol/L	F	99.0	108	99.4	108	99.8	108	99.0	108	98.9	108
TSH	mIU/L	M	0.60	4.95	0.62	4.82	0.66	4.34	0.58	5.09	0.57	5.19
TSH	mIU/L	F	0.64	6.21	0.68	6.03	0.68	5.70	0.62	6.34	0.63	6.32
FT4	ng/dL	M	0.62	1.14	0.60	1.10	0.59	1.07	0.61	1.17	0.61	1.17
FT4	ng/dL	F	0.60	1.15	0.59	1.09	0.59	1.08	0.59	1.16	0.59	1.17
FT3	pg/mL	M	2.46	4.16	2.59	4.27	2.54	4.21	2.55	4.30	2.59	4.28
FT3	pg/mL	F	2.39	3.97	2.46	4.04	2.49	4.07	2.44	4.15	2.44	4.11

LL, Lower Limit; UL, Upper Limit; TP, Total Protein; ALT, Alanine Aminotransferase; AST, Aspartate Aminotransferase; GGT, Gamma-Glutamyl Transferase; LDH, Lactate Dehydrogenase; TB, Total Bilirubin; Ca, Calcium; Mg, Magnesium; P, Phosphorus; TSH, Thyroid Stimulating Hormone; FT4, Free Thyroxine; FT3, Free Triiodothyronine. Green: accept = within tolerance; yellow: check = slightly increase/decreased; red: reject = markedly increase/decreased.

**Table 4:** Percentage or absolute differences between the reflimR-estimated reference limits and those derived from other indirect methods.

Analyte	Sex	reflimR-refineR		reflimR-KOSMIC		reflimR-Hoffmann		reflimR-Bhattacharya		Acceptable limits^a^
		LL	UL	LL	UL	LL	UL	LL	UL	
Glucose	M	−0.13	1.71	0.92	1.71	0.13	0.00	0.26	0.85	8.00%
Glucose	F	−3.24	0.90	−2.74	1.82	−2.74	−0.88	−2.74	−1.75	8.00%
Albumin	M	−0.76	0.39	−1.01	0.39	1.03	0.00	−0.51	0.19	8.00%
Albumin	F	−0.77	−0.20	−1.27	0.41	0.78	−0.40	0.00	0.00	8.00%
TP	M	−0.16	0.12	−1.24	−0.24	1.44	−0.36	−0.31	0.12	8.00%
TP	F	−0.47	−0.24	−0.63	0.36	0.48	−0.12	0.16	0.12	8.00%
ALT	M	−3.94	**16.9**	1.10	**16.2**	−4.60	4.14	−2.35	4.79	15.0%
ALT	F	−0.82	8.57	−3.05	2.36	−1.35	−3.80	−3.95	−3.18	15.0%
AST	M	−1.45	3.13	0.74	7.10	−2.16	−1.36	−0.73	3.43	15.0%
AST	F	−2.31	7.17	1.60	8.28	−2.31	−0.32	0.79	1.95	15.0%
GGT	M	−2.75	6.24	0.95	**18.5**	−6.19	−0.31	−0.93	0.15	15.0%
GGT	F	−1.48	4.04	5.40	6.69	−7.62	2.76	0.88	3.72	15.0%
LDH	M	0.85	0.00	−0.83	4.35	0.85	−2.04	0.85	−0.41	15.0%
LDH	F	0.00	0.00	0.00	2.46	0.00	−1.57	0.00	0.00	15.0%
TB	M	3.33	2.82	−3.13	**20.7**	10.7	13.2	3.33	2.82	20.0%
TB	F	0.00	10.9	13.0	10.9	8.33	6.25	0.00	−6.42	20.0%
Ca	M	0.01	0.00	−0.20	0.30	0.03	0.00	0.11	0.00	± 1.00 mg/dL
Ca	F	0.11	−0.20	0.11	−0.10	0.17	−0.20	0.19	−0.10	± 1.00 mg/dL
Mg	M	1.78	−0.44	0.58	2.71	3.61	−2.58	6.83	0.00	15.0%
Mg	F	4.29	−0.44	1.19	0.90	4.94	−1.75	7.59	0.45	15.0%
P	M	5.02	4.00	−1.18	3.77	3.72	1.08	5.02	0.86	10.0%
P	F	−2.15	−4.17	0.74	−1.71	3.41	−1.50	6.23	−0.86	10.0%
Urea	M	−0.54	1.73	−1.60	3.73	0.54	−1.68	1.09	−0.94	9.00%
Urea	F	0.68	0.41	0.00	3.82	0.68	−1.01	1.38	−0.81	9.00%
Creatinine	M	0.00	1.67	1.52	1.67	3.08	0.00	1.52	0.00	10.0%
Creatinine	F	−1.96	3.30	0.00	5.62	0.00	−2.08	0.00	0.00	10.0%
Sodium	M	0.00	0.00	−1.00	0.00	0.00	0.00	0.00	0.00	± 4.00 mmol/L
Sodium	F	0.00	0.00	0.00	0.00	0.00	0.00	0.00	0.00	± 4.00 mmol/L
Potassium	M	0.01	−0.07	0.02	0.06	0.09	−0.13	0.10	0.02	± 0.30 mmol/L
Potassium	F	0.02	0.04	−0.01	0.10	0.05	−0.11	0.14	−0.08	± 0.30 mmol/L
Chloride	M	−0.61	0.00	−2.00	1.89	−0.20	0.00	−0.20	0.00	5.00%
Chloride	F	−0.40	0.00	−0.80	0.00	0.00	0.00	0.10	0.00	5.00%
TSH	M	−3.23	2.70	−9.09	14.1	3.45	−2.75	5.26	−4.62	20.0%
TSH	F	−5.88	2.99	−5.88	8.95	3.23	−2.05	1.59	−1.74	20.0%
FT4	M	3.33	3.64	5.08	6.54	1.64	−2.56	1.64	−2.56	15.0%
FT4	F	1.69	5.50	1.69	6.48	1.69	−0.86	1.69	−1.71	15.0%
FT3	M	−5.02	−2.58	−3.15	−1.19	−3.53	−3.26	−5.02	−2.80	20.0%*
FT3	F	−2.85	−1.73	−4.02	−2.46	−2.05	−4.34	−2.05	−3.41	20.0%*

The percentage difference values indicated in bold exceeded the acceptable limits. LL, Lower Limit; UL, Upper Limit; TP, Total Protein; ALT, Alanine Aminotransferase; AST, Aspartate Aminotransferase; GGT, Gamma-Glutamyl Transferase; LDH, Lactate Dehydrogenase; TB, Total Bilirubin; Ca, Calcium; Mg, Magnesium; P, Phosphorus; TSH, Thyroid Stimulating Hormone; FT4, Free Thyroxine; FT3, Free Triiodothyronine. ^a^ Acceptable limits were defined according to the Clinical Laboratory Improvement Amendments (CLIA).

## Discussion

In this study, we applied the reflimR algorithm, a state-of-the-art indirect method for the verification of RIs, to real-world data and compared it with other methods and IFU RIs. Although the CLSI C28-A3c guideline considers it sufficient for no more than two out of 20 healthy individuals to fall outside the proposed RI during verification [2], this approach may fail to identify RIs that are excessively wide. Therefore, the risk of accepting an excessively wide RI is not eliminated by this method, and the representativeness and reproducibility of this alternative approach are limited [12,13]. Unlike the guideline-based approach [2], reflimR applies a more stringent evaluation of the proposed RIs, thereby preventing the erroneous acceptance of inappropriate limits [10].

Using the reflimR method, the modified method was fully automated in R and allowed calculations to be performed within milliseconds. The significantly higher speed of the reflimR method compared to that of refineR is an advantage for routine laboratory applications. Fast results are important, particularly when confidence intervals are calculated using simulations or bootstrap techniques. The reflimR package incorporates a color scheme (traffic light colors) to illustrate how well the estimated RIs align with the predefined limits of the laboratory. These colors (green, yellow, and red) are not subjective because they are determined based on the permissible uncertainty (tolerance limits) of laboratory results [10].

For the majority of the LRLs and URLs (40/76) of the analytes evaluated, the RIs predicted by reflimR were similar to and consistent with the IFU RIs. For some analytes, slight or marked differences in RIs were observed. In our study population, the URLs for glucose, AST, total bilirubin, and urea tests in men and AST, LDH, total bilirubin, and urea tests in women significantly exceeded the tolerance limits determined using the IFU or literature limits. Additionally, the LRLs for albumin, AST, potassium, and TSH in women and albumin, potassium, and TSH in men significantly exceeded the tolerance limits determined using the IFU limits (Table 2). In the study by Hoffman et al., while URLs for ALT, AST, and creatinine in men and URLs for albumin and bilirubin tests in women significantly exceeded the tolerance limits, the tests that significantly exceeded the reflimR algorithm for LRLs were determined as AST, bilirubin, and creatinine in men and AST and creatinine tests in women [10]. This may be due to fundamental differences (e.g., ethnicity, sex and age distribution, time period, and measurement location) between the IFU studies and our population analyzed using the indirect method.

The RIs for biochemical tests performed using the AU480 analyzer in Ghanaian adults were calculated using a parametric method. The RIs calculated in our study for total protein (65−84 g/L), creatinine (male: 0.66–1.23 mg/dL, female: 0.45–0.93 mg/dL), sodium (136−143 mmol/L), and chloride (99−108 mmol/L) were highly consistent with the limits obtained in this study [14]. Although the LRLs (percentage difference: 11.7% in males and 11.1% in females) calculated for albumin in our study could not be verified when compared with the IFU values, similar LRLs were obtained in studies conducted in Ghana (2.89% in males and 2.37% in females) [14], Kenya (−2.25% in males and 2.37% in females) [15], and Russia (0.26% in males and −0.26% in females) [16] using the same manufacturer’s assay, demonstrating substantial agreement with our findings. For glucose, the URL (12.3% in males) calculated in our study was markedly higher than that reported by the manufacturer. Compared with the URL reported in the Ghanaian study [14], our URLs were also higher (11.2% in males and 4.67% in females). Notably, in Kenya [15] higher glucose URL for males (9.85%) were observed, particularly in individuals aged > 45 years. Compared with the URLs reported in the Russian study, our URLs demonstrated better agreement, especially in participants older than 45 years, with percentage differences of 1.71% for males and 0.00% for females [16]. As our study population had median and quartile values of 52 (38–62) for men and 49 (35–60) for women without age stratification, the observed elevation may be attributed to age-related variations. The URLs for total bilirubin calculated in our study differed markedly from the manufacturer’s claimed values, being higher in males (21.7%) and lower in females (−15.0%). Furthermore, studies conducted in Ghana (30.8% in males and 27.1% in females) [14] and Kenya (41.8% in males and 35.4% in females) [15] have reported markedly higher URL values than those found in our study. Consistent with our findings, these studies also demonstrated higher URLs in males compared than in females. The RIs for total bilirubin reported in the Kenyan study were nearly twice as high as those reported in studies conducted outside the African continent. This may be attributed to the higher prevalence of Gilbert syndrome, the most common genetic cause of asymptomatic unconjugated hyperbilirubinemia in Kenya [15]. A study conducted in Russia attributed this finding to the presence of Gilbert syndrome [16]. Total bilirubin levels exhibited sex-related differences, with males showing higher URLs. The prevalence of Gilbert syndrome in Europe is estimated to be approximately 3–6%, with a marked male predominance, reflected by a male-to-female ratio of 4:1 [17]. Genetic studies conducted in Türkiye have shown that Gilbert syndrome is largely associated with the A(TA)7TAA (UGT1A1*28) polymorphism in the promoter region of the UGT1A1 gene and that this variant is predominant in the population. Additionally, other rare UGT1A1 sequence alterations, such as c.1091C > T (p.Pro365Leu) and c.880_893delinsA, have been reported to affect bilirubin metabolism and contribute to this phenotype [18]. When the URLs were compared with previously published reference intervals from Türkiye, the percentage differences were 4.29% for men and –15.7% for women on the Roche platform [19] and 3.5% for men and 9.7% for women on the Abbott platform [11]. All URL percentage differences were within the allowable limits (20%) defined by the CLIA. For urea, the URLs (22.8% in males, 13.7% in females) calculated in our study was markedly higher than those reported by the manufacturer. The URLs for urea calculated in our study were markedly higher (55.3% in males, 43.8% in females) than those reported in a Ghanaian study [14]. In contrast, the urea RIs estimated in the Kenyan study [15] were lower than those reported in Türkiye [11] and Saudi Arabia [20]. Urea RIs estimated in Türkiye (−22.2%) [11] and Saudi Arabia (−20.6%) [20] were markedly lower than those found in our study for males. However, in females, the URLs reported for individuals aged over 50 years in the Turkish study (2.30%) [11] and over 45 years in the Russian study (3.17%) [16] showed better agreement with the URLs established in our study. These differences may be attributable to variations in protein-rich dietary intake. In a Chinese study using Beckman Coulter^®^ AU5800, albumin, total bilirubin, ALT, and GGT levels were generally higher in males than in females, likely due to factors such as greater muscle mass, higher alcohol consumption, and increased obesity prevalence in men. These sex-related physiological and behavioral differences may account for the observed disparities in liver enzyme, bilirubin, and protein levels [21]. Although studies from Ghana [14] and Russia [16] were similar to those from the IFU in terms of the potassium test, the LRL values obtained in our study were higher than the limits (0.30 mmol/L in males, 0.32 mmol/L in females) reported in both countries [14,16]. When the LRLs were compared with previously published reference intervals from Türkiye, the absolute differences on the Roche platform were 0.10 mmol/L for men and 0.02 mmol/L for women [19], whereas on the Abbott platform they were 0.10 mmol/L for men and 0.12 mmol/L for women [11]. Notably, all absolute differences in LRLs remained within the allowable limit of 0.30 mmol/L. Although our LRL for chloride was slightly lower than the IFU value, our limits were identical to those reported (1.01% in males, 0.00% in females) in the Ghanaian [14] and Russian [16] studies. This study [11] yielded potassium and chloride LRL values that were higher and lower, respectively, than those of IFU, which is in agreement with our findings. For ALT, the value obtained for females was very close to that reported in the Kenyan [15] and Türkiye [19] (%1.33 for both studies), whereas the URL for males was lower than the Kenyan and Türkiye values (−8.55% for both studies). Regarding AST, the URLs derived in our study were consistent with those reported in studies conducted in Kenya (9.50% in males, 8.28% in females; acceptable limits: 15%) [15]. As the manufacturer-provided LDH RIs did not include LRLs, comparison with reflimR results was not possible and literature-based limits [11] were used instead. In females, the LDH URL was higher than those reported in the literature but showed good agreement (3.23% in males, 1.21% in females) with the IFU value.

Agaravatt et al. reported higher URLs for TSH using both the KOSMIC and refineR algorithms, whereas the Hoffman method detected lower URLs. The LRLs of TSH and URLs and LRLs for FT4 and FT3 showed a good correlation with IFU using the Hoffman, KOSMIC, and refineR methods [22]. In our study, while LRL levels were significantly higher in both sexes for TSH than for IFU, we detected slightly higher values for TSH URL only in women and slightly higher values for FT3 URL only in men. In another study [12], similar to our findings, both the directly and indirectly estimated LRL values for the TSH test on the Beckman Coulter^®^ DxI analyzer were found to be higher than the manufacturer’s claimed LRL of 0.38. The study generally indicated that manufacturer-provided RIs for TSH, particularly those of Abbott^®^, Roche^®^, and Beckman Coulter^®^, exhibited inappropriate URLs. However, it was noted that the Beckman Coulter^®^ analyzer showed different and broader limits for TSH compared to the manufacturer’s claims [12]. Although TSH LRL verification was unsuccessful, the values obtained in the Kenyan study were nearly identical to the limits we calculated for males, indicating that the LRL values were higher than those reported in the IFU. In the same study, the FT3 and FT4 limits were consistent with the IFU, which is similar to our findings [15]. However, in our study, the URL values for females were higher than those for males, which may be attributed to the higher prevalence of subclinical hypothyroidism among women [23].

The URL calculated using the reflimR algorithm for ALT (absolute difference: 7.30 U/L, percentage difference: 16.9%; acceptable limit: 15.0%) in males was markedly higher than that calculated using the refineR algorithm. The URLs calculated using the reflimR algorithm were markedly higher for GGT (absolute difference: 10.1 U/L, percentage difference: 18.5%; acceptable limit: 15.0%) and total bilirubin (absolute difference: 0.25 mg/dL, percentage difference: 20.7%; acceptable limit: 20.0%) levels in males than those calculated using the KOSMIC algorithm. According to the permissible uncertainty algorithm of reflimR, these parameters differed between the indirect methods (Table 3). Moreover, when evaluated against CLIA target values, they exceeded the allowable limits, indicating clinically meaningful differences (Table 4). This observation may be attributable to the underlying mathematical assumptions of the indirect algorithms as well as the data distribution (right-skewed) characteristics. Indirect methods utilize real-world data generated continuously throughout the patient care process, encompassing both non-pathological (physiological) and pathological test results. For different analytes, discrepancies between indirect methods may be observed as the pathological data size changes depending on the study population [9]. Reference limit estimation becomes the most challenging when physiological and abnormal test results overlap substantially, particularly when abnormal distributions are centered near the true reference limits, leading to increasing deviations, particularly when the proportion of abnormal values is ≥ 20%. In simulations and real-world data, it was found that the difference between the predicted reference limits and the actual values was more pronounced at the URLs than at the LRLs. In simulations performed using KOSMIC, it has been observed that the URLs calculated for GGT are estimated to be much lower than those for other analytes, such as hemoglobin and TSH [8]. Parameters such as liver enzymes (ALT and GGT) and total bilirubin inherently exhibit right-skewed distributions owing to their biological characteristics and show substantial overlap between healthy and pathological populations. In such challenging datasets, modern indirect methods such as refineR and KOSMIC employ complex statistical models to identify the “pure” physiological distribution; refineR relies on regularized maximum likelihood optimization combined with Box–Cox transformations [9], whereas KOSMIC aims to minimize the Kolmogorov–Smirnov (KS) distance between empirical and theoretical distributions following Box–Cox transformation [8]. In contrast, reflimR follows a different methodological approach, combining modified boxplot-based iterative truncation with a regression strategy that focuses on the central linear region of the normal Q–Q plot [10]. For GGT, the high number of slightly elevated results that may occur because of alcohol use, overweight participants, and medication use may introduce bias into the indirect methods used to separate the pathological fraction from the healthy distribution [24,25] and consequently may be excluded in an overly aggressive manner (over-truncation). This may cause the model to constrain the healthy population into a narrower range, thereby leading to underestimation of the URLs compared to reflimR [8,9]. Considering all methods, it would be more appropriate to determine the RIs using analyte-specific approaches, especially for ALT, GGT, and total bilirubin tests, according to our study. The use of personalized RIs with population-based RIs may be useful in clearly distinguishing pathological from non-pathological boundaries [26], and will need to be determined by the scientific community in future studies.

To the best of our knowledge, this is the first external validation of reflimR using a large routine dataset obtained from a Turkish adult population, and it provides a new perspective on its applicability in different demographic and epidemiological contexts. Second, by performing a systematic comparison of five widely used indirect algorithms (reflimR, refineR, KOSMIC, Hoffmann, and Bhattacharya), we enabled an evaluation of methodological concordance for reflimR.

This study has some limitations. Initially, this was a single-center study, which might lead to selection bias and could impact the generalizability of the RIs. Additionally, there may have been incomplete exclusion criteria that could have affected the outcomes. This study exclusively included individuals aged 18–75 years, thereby excluding both the pediatric population and those over 75 years of age. Additionally, the verification and calculation of RIs were performed without employing age partitioning.

## Conclusion

The reflimR algorithm may be helpful for RI verification with intuitive color-coded result interpretation and minimal data requirements for estimates. Fifteen of the 76 RI limits were rejected for RI verification. This may be due to fundamental differences (e.g., ethnicity, sex and age distribution, time period, and measurement location) between the IFU studies and our population analyzed using the indirect method. Comparison of reflimR with other indirect methods generally produced concordant results, except for ALT, GGT, and total bilirubin tests. The differences in the RIs obtained from various indirect methods may be attributed to the data distribution and variations in the computational approaches of the algorithms.

## Supporting information

S1 File Study dataset. Anonymized dataset used for all statistical analyses in this study.
(XLSX)
